# Mild-to-moderate COVID-19 impact on the cardiorespiratory fitness in young and middle-aged populations

**DOI:** 10.1590/1414-431X2022e12118

**Published:** 2022-07-13

**Authors:** G.D. Back, M.R. Oliveira, P.F. Camargo, C.L. Goulart, C.R. Oliveira, K.W. Wende, J.C. Bonjorno, R.F. Arbex, F.R. Caruso, R. Arena, A. Borghi-Silva

**Affiliations:** 1 Laboratório de Fisioterapia Cardiopulmonar, Departamento de Fisioterapia, Universidade Federal de São Carlos, São Carlos, SP, Brasil; 2 Department of Physiotherapy, College of Applied Sciences, University of Illinois at Chicago, Chicago, IL, USA; 3 Healthy Living for Pandemic Event Protection (HL-PIVOT) Network, Chicago, IL, USA

**Keywords:** COVID-19, Survivors, Pulmonary function, Cardiorespiratory fitness, Cardiopulmonary testing

## Abstract

The goal of the present study was to compare pulmonary function test (PFT) and cardiopulmonary exercise test (CPET) performance in COVID-19 survivors with a control group (CG). This was a cross-sectional study. Patients diagnosed with COVID-19, without severe signs and symptoms, were evaluated one month after the infection. Healthy volunteers matched for sex and age constituted the control group. All volunteers underwent the following assessments: i) clinical evaluation, ii) PTF; and iii) CPET on a cycle ergometer. Metabolic variables were measured by the CareFusion Oxycon Mobile device. In addition, heart rate responses, peak systolic and diastolic blood pressure, and perceived exertion were recorded. Twenty-nine patients with COVID-19 and 18 healthy control subjects were evaluated. Surviving patients of COVID-19 had a mean age of 40 years and had higher body mass index and persistent symptoms compared to the CG (P<0.05), but patients with COVID-19 had more comorbidities, number of medications, and greater impairment of lung function (P<0.05). Regarding CPET, patients surviving COVID-19 had reduced peak workload, oxygen uptake (*V̇*O_2_), carbon dioxide output (*V̇*CO_2_), circulatory power (CP), and end-tidal pressure for carbon dioxide (*P*_ET_CO_2_) (P<0.05). Additionally, survivors had depressed chronotropic and ventilatory responses, low peak oxygen saturation, and greater muscle fatigue (P<0.05) compared to CG_._ Despite not showing signs and symptoms of severe disease during infection, adult survivors had losses of lung function and cardiorespiratory capacity one month after recovery from COVID-19. In addition, cardiovascular, ventilatory, and lower limb fatigue responses were the main exercise limitations.

## Introduction

Since the emergence of a new coronavirus, the severe acute respiratory syndrome coronavirus 2 (SARS-CoV-2) in late 2019 in the city of Wuhan, China, the coronavirus disease 2019 (COVID-19) (1) has infected more than 334 million people globally since January 2022 (2). In Brazil, there are more than 22 million individuals who have recovered from COVID-19, a significant percentage of whom have persisting symptoms after infection (2,3). Among the persistent and already known sequelae, reduction in cardiorespiratory fitness (CRF) and pulmonary function, exertional dyspnea, and muscle fatigue are common. These clinical symptoms are associated with lesions in multiple organs, especially in patients hospitalized with moderate and critical infections, of advanced age, and who remained hospitalized for a prolonged time (4,5). However, data on the impact of milder manifestations of COVID-19 in the adult population needs further investigation.

Bellan et al. (6) found that a significant proportion of COVID-19 survivors had respiratory and functional impairment 4 months after hospital discharge. They stated that more than half of the population studied still had a diffusing capacity of the lungs for carbon monoxide (DLCO) below 80% of the predicted value (6). Huang et al. (7) also reported that more than 50% of the patients had DLCO below 80% of predicted 30 days after hospital discharge. Furthermore, Huang et al. (5) reported that COVID-19 survivors had persistent fatigue and muscle weakness 6 months after infection. Raman et al. (4) also found that a significant proportion of patients reported symptoms of shortness of breath, fatigue, and limited exercise capacity 2 to 3 months after hospital discharge. These studies included patients with varying COVID-19 severity and different ages between groups. This is a limitation in the literature, as lung function and CRF are directly influenced by these factors (8).

Understandably, there are ongoing efforts to understand the repercussions of COVID-19 infection with more severe manifestations, especially in hospitalized patients (9). A similar line of inquiry is needed in patients who did not present with severe disease. Despite not showing signs of severe disease in the acute infection, it is known that these subjects may have persistent symptoms and important limitations (10). Such information is important to better understand this emerging patient group and, among other things, optimize rehabilitation approaches to restore lost function and quality of life.

Cardiopulmonary exercise testing (CPET) is the gold-standard approach to assessing CRF and offers enormous potential in identifying pathophysiological factors and prognostic characteristics of a disease state (11). CPET can provide an accurate understanding of physiological capabilities and limitations, even in the face of the current pandemic, and it is necessary to highlight the importance of continuing the diagnostic and prognostic assessment of cardiovascular diseases and the evaluation of therapeutic efficacy in post-COVID-19 patients (12).

To date, studies used CPET after 1 to 3 months of COVID-19 infection and found that patients achieve a lower peak oxygen consumption (*V̇*O_2_) and oxygen uptake efficiency slope (OUES) and a higher minute ventilation/carbon dioxide production (*V̇*_E_/*V̇*CO_2_) slope, especially patients presenting with severe COVID-19 (4,13-15). Other studies only used assessments of submaximal cardiorespiratory capacity, such as the 6-minute walk test (5,7,16), also indicating limitations through shorter walking distances, below the predicted values adjusted for age, in patients with severe COVID-19.

At this point, studies are needed to understand the sequelae of COVID-19 from cardiorespiratory assessments in mild-to-moderate COVID-19 in adults. Therefore, the aim of the present study was to compare complete lung function and CPET performance in COVID-19 survivors with a control group. Our hypothesis was that patients who tested positive for COVID-19 have a poorer lung capacity, impaired lung function, and a decline in CRF one month after diagnosis compared to the control group. Furthermore, we believe that patients with COVID-19 who present with milder symptoms also have limitations in lung function and CRF.

## Material and Methods

### Study design and ethical approval

This cross-sectional study was designed following the recommendations of the STROBE declaration. This study met the ethical guidelines of the Declaration of Helsinki (17) and was approved by the Ethics Committee (Federal University of São Carlos) (protocol number: 32408720.5.0000.5504). Volunteers were recruited at the UFSCar University Hospital and at the Santa Casa de Misericórdia de São Carlos from June to November 2020. COVID-19 survivors were screened one month after infection and were invited to undergo CPET (September of 2020 to March 2021); they were contacted by telephone to schedule evaluations. Patients were selected and recruited by members of the research team who visited the hospital weekly. All volunteers signed an informed consent to participate in the study.

### Subjects

Subjects of both sexes were included if they were: 1) positive for COVID-19 based on nasal swab real-time reverse transcriptase-polymerase chain reaction and 2) aged between 18 and 60 years. The severity of the acute illness was defined by the provisional clinical guidance of the World Health Organization (WHO) (18). Mild cases were classified as: 1) flu syndrome with mild symptoms (no dyspnea or signs of severity, without evidence of viral pneumonia or hypoxia); 2) absence of decompensated comorbidity without the need for hospitalization; and 3) home isolation for at least 10 days. Moderate cases were defined as: 1) moderate symptoms with clinical signs of pneumonia (fever, cough, dyspnea, rapid breathing that required stabilization and admission to the ward, non-invasive ventilatory support); 2) no signs of severe pneumonia, including SpO_2_ ≥90% on room air; 3) involvement of ≤50% of the lung parenchyma on computed tomography; 4) hospitalization ≤10 days; and 5) respiratory and motor physiotherapy at least once a day. The control group (CG) consisted of healthy subjects who sought the aforementioned health services for suspected disease and tested negative for COVID-19, according to WHO guidelines (18). After negative diagnosis, volunteers without comorbidities, who did not use controlled medications, non-smokers, and matched by age and sex were selected for the CG.

Exclusion criteria were: 1) absence of informed consent; 2) over 60 years of age; 3) being hospitalized for less than 72 h or more than 10 days; 4) severe forms of COVID-19 in the initial phase of hospitalization with a respiratory rate >30 breaths/min; 5) severe breathing difficulty; 5) SpO_2_ <90% on room air that culminated in sedation and intubation with invasive mechanical ventilation and admission to the intensive care unit (ICU); 6) altered mental status, rapid heart rate, weak pulse, cold extremities or cyanosis, skin spots, positive for coagulopathy, thrombocytopenia, acidosis, or high lactate; 7) acute respiratory exacerbation within 4 weeks before enrollment; and 8) use of home oxygen, use of illicit drugs or alcoholics, pregnant women, dementia, and presence of medical conditions contraindicating CPET (acute or unstable cardiorespiratory conditions, musculoskeletal impairment that can compromise performance in exercise) (19). The CG did not have cardiovascular, respiratory, or metabolic comorbidities that could interfere with the study results.

### Experimental procedures

**Figure 1:**
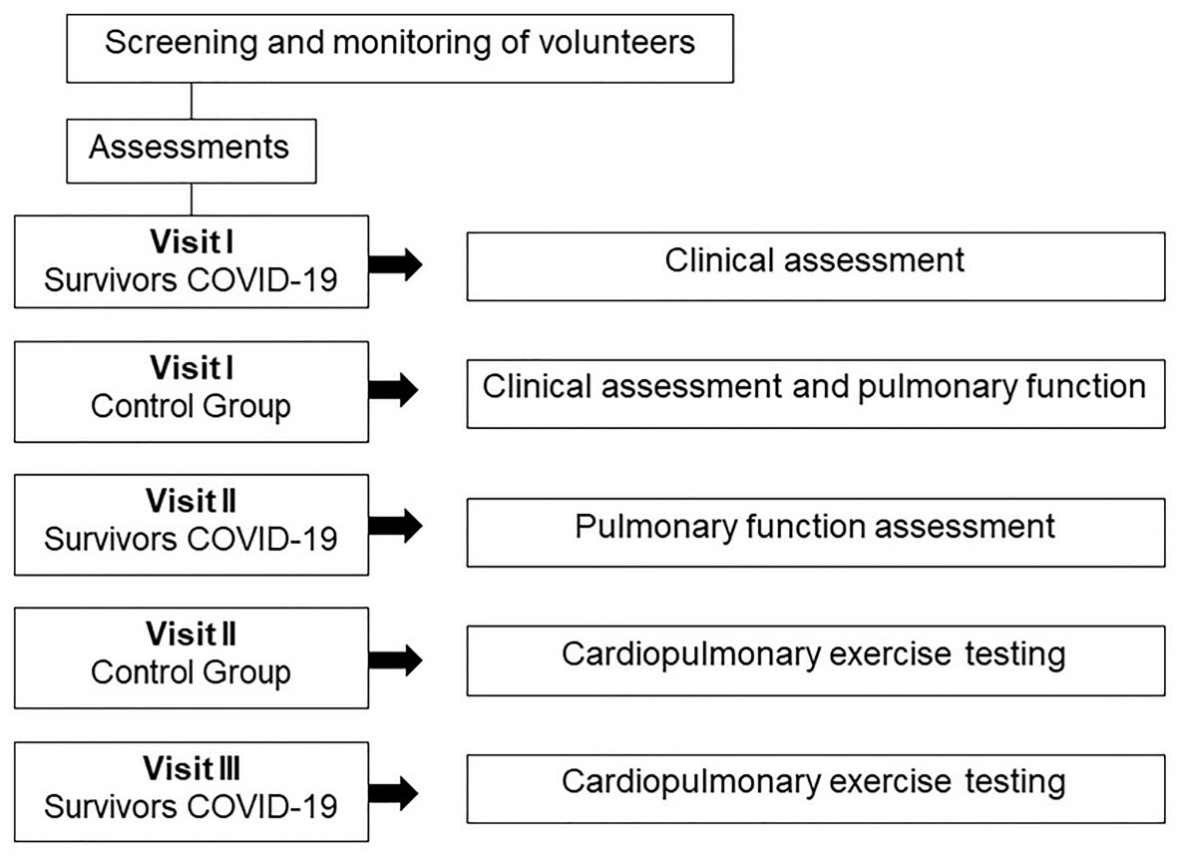
Evaluation flow chart.

Subjects were selected and recruited by members of the research team who visited the hospital weekly. Hospitalized patients were followed during hospitalization and after hospital discharge through telephone calls on the 20th day. Survivors in home quarantine and the CG were allocated from a list of test results provided by the hospitals. Patients who survived COVID-19 underwent a clinical evaluation divided into three consultations and the CG was evaluated in two consultations and all evaluations were carried out with an interval of 48 hours between each laboratory visit (Figure 1). Research team members were properly equipped with personal protective equipment (PPE), including a waterproof apron, goggles, latex gloves, N95 mask, disposable mask, disposable cap, and face shield. In addition, the entire physical space was adapted according to current recommendations (18).

### Evaluations and measurements

Patients who recovered from COVID-19 were evaluated one month after the acute phase of infection. All volunteers were contacted by phone 48 h before the assessment. At the time of screening, questions were asked about symptoms of COVID-19 in the last 10 days. In addition, volunteers were instructed to wear a mask and to adhere to the recommendations for social distancing on the way to the laboratory. The following evaluations were carried out.

### Clinical evaluation

Clinical data including age, sex, weight, height, race, information on past medical history, comorbidities, persistent symptoms, smoking, and medication use were collected to characterize the sample.

### Pulmonary function

Static and dynamic lung volumes, lung diffusion capacity, and respiratory muscle strength were measured using complete lung function by the MasterScreen™ Body Plethysmograph (Mijnhardt/Jäguer, Germany). Disposable mouthpieces with filters (MicroGard^®^ II, filters) 99.999% effective against cross contamination with virus and bacteria were used. Additionally, for each subject, the plethysmograph was sanitized according to the manufacturer's instructions. The variables analyzed were: tidal volume (TV), forced expiratory volume in 1s (FEV_1_), forced vital capacity (FVC), total lung capacity (TLC), functional residual capacity (FRC), inspiratory capacity (IC), carbon monoxide diffusing capacity (DLCO), carbon monoxide transfer coefficient (KCO), maximum inspiratory pressure (MIP), and maximum expiratory pressure (MEP). Spirometry was performed according to the recommendations of the American Thoracic Society/European Respiratory Society (20) and the results were compared to previously described reference values (21).

### Cardiopulmonary exercise testing

All tests were performed according to the American College of Cardiology and American Heart Association Guidelines (22), supervised by a physician and two previously trained physical therapists. All exercise tests were performed on a cycle ergometer with electromagnetic braking (Corival Recumbent, Medical Graphics Corp., USA). Metabolic gases were evaluated using the Oxycon Mobile respiratory gas analyzer (Mijnhardt/Jäger) in a ventilated and sterile room.

The protocol consisted of the following: 1) 5-min rest period while sitting on the cycle ergometer; 2) 1-min exercise at free-wheel and 60 rotations per minute (rpm); 3) incremental phase with an increase of 5-20 W/min (ramp protocol); 4) 1-min active recovery at free-wheel; and 5) 5-min passive recovery resting in sitting position. A twelve-lead electrocardiogram (ECG) was continuously monitored throughout the test (WinCardio, Micromed, Brazil).

The test was finished when subjects were pedaling at their maximum possible effort level (physical exhaustion) and reported at least 2 of the following criteria: 1) age-predicted maximal HR (220 - [age]); 2) general/leg fatigue or dyspnea; 3) angina or electrocardiographic evidence of ischemia or malignant arrhythmia (ventricular tachyarrhythmia, ventricular fibrillation, bigeminism); or 4) the inability to maintain a pedaling rate of 60 rpm for 30 s (19). The load prescription (W) was based on the recommendation of the American College of Sports Medicine, where load (W) = [(height - age) × 12] - [(150 + 6 × weight)] / 100, and according to the reported exercise tolerance (23).

### Ventilatory and hemodynamic measurements during CPET

During CPET the following parameters were measured: workload (WR) (watts), peak *V̇*O_2_ (mL·kg^−1^·min^−1^), *V̇*O_2_ (mL/min), *V̇*CO_2_ (mL/min), the *V̇*_E_/*V̇*CO_2_ slope, HR peak (bpm), and the OUES (24). Circulatory power (CP) was obtained through the product of peak *V̇*O_2_ and peak systolic blood pressure (25), and ventilatory power (VP) was calculated by dividing peak systolic blood pressure by the *V̇*_E_/*V̇*CO_2_ slope (26). O_2_ pulse was calculated using the product of peak *V̇*O_2_ and peak HR. *V̇*O_2_/WR was determined by the relationship between maximal workload obtained and *V̇*O_2_ peak, and peak and rest of systolic and diastolic blood pressures (SBP and DBP, mmHg) (27). The arterial oxygen saturation was measured non-invasively by pulse oximetry (SpO_2_, %). The Borg dyspnea scale was used to assess lower limb muscle fatigue and shortness of breath at the peak of the test (28).

### Statistical analysis

Data are reported as means±SD for continuous variables and as percentages for categorical variables. The Shapiro-Wilk test was used to verify data distribution. Student's *t*-test and the chi-squared test were used to compare the groups of survivors from COVID-19 and the CG.

Pearson's correlation analysis was performed to investigate correlations between variables. The magnitude of the correlations was determined considering the following classification scheme for r values: ≤0.35 low or weak; 0.36 to ≤0.67 moderate; ≥0.68 to ≤0.89 strong or high; ≥0.9 very high; and perfect: 1 (29). Simple linear regression was performed to determine the ability of DLCO (mL) and Δ*P*_ET_CO_2_ (mmHg) to predict *V̇*O_2_ (mL·kg^−1^·min^−1^) in the COVID-19 survivor group. All statistical analyses were performed using the Statistical Package for Social Sciences (SPSS) 20.0 program (IBM, USA). A P value ≤0.05 was considered statistically significant for all tests. To analyze the power of the study sample, a *post hoc* calculation of the power of the sample was performed for the results of the comparison between the COVID-19 survivor groups (n=29) and CG (n=18). Two-way independent *t*-test was used, with α=0.05. The effect size was determined by the mean of each group for the variable *V̇*O_2pred_ (%) (COVID-19 survivors: 61% and CG: 86%) and standard deviation of the groups. Thus, the value of sample power was (1-β)=0.999, considered a large sample power. The sample size calculation was performed using G*Power 3.1 (University of Dusseldorf, Germany).

## Results

**Figure 2:**
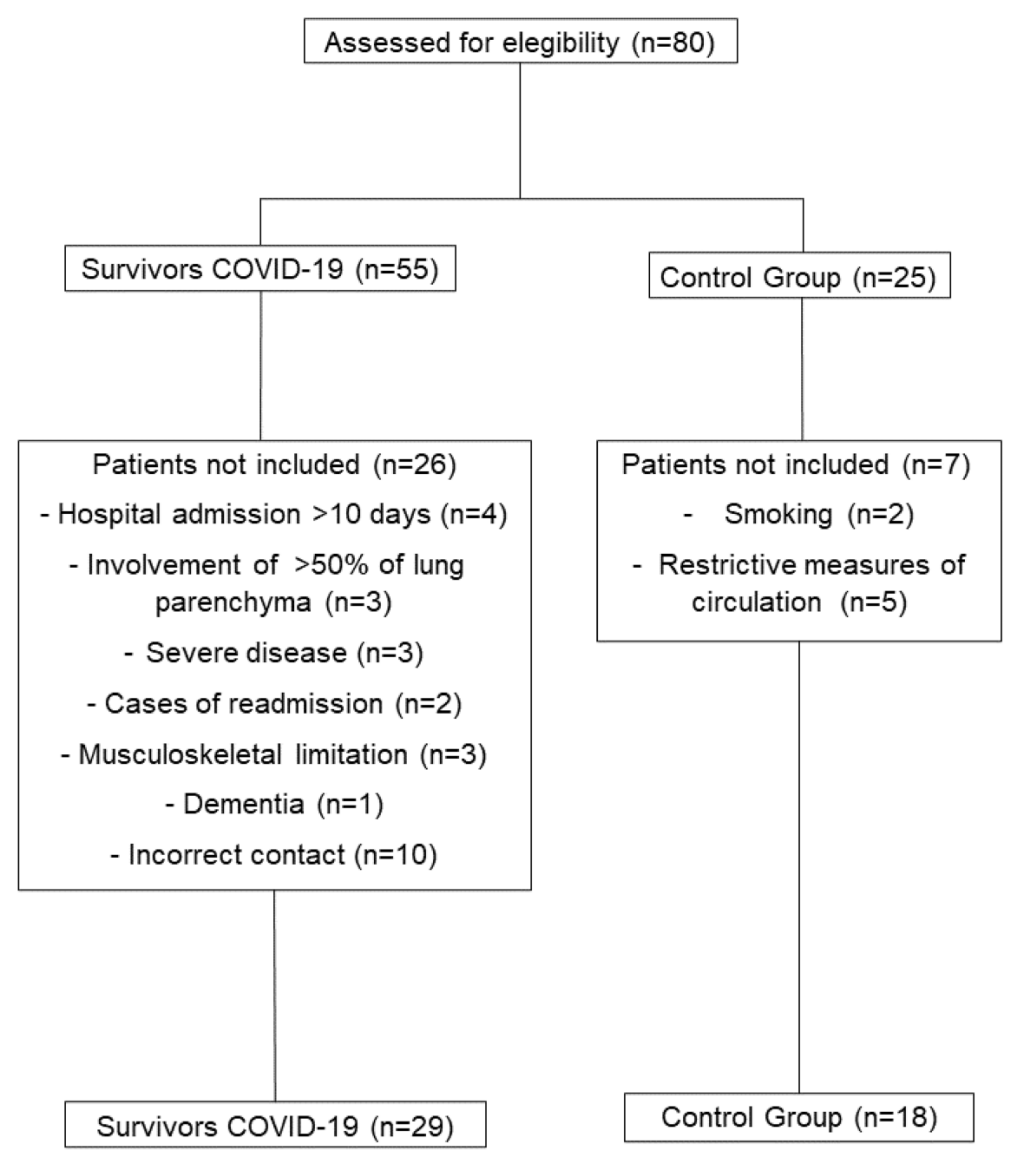
Study flow chart.

Eighty patients were initially recruited after stratification and divided into two groups: COVID-19 survivors (n=55) and CG (n=25). The reasons for exclusion for COVID-19 survivors were: 1) four patients with a hospital stay of more than 10 days; 2) three patients had pulmonary impairment greater than 50% on computed tomography; 3) three critical cases of COVID-19; 4) two patients were readmitted to the hospital for other diseases; 5) three patients had musculoskeletal limitations; 6) one case of dementia after hospital discharge; and 7) 10 patients were excluded for not answering the phone call. Regarding the CG, given the alarming situation of the pandemic in Brazil, five volunteers did not undergo the assessments due to restrictive measures and two smoking volunteers at the time of the assessment were excluded. In total, 29 COVID-19 survivors were evaluated. Additionally, 18 healthy volunteers matched by sex and age were allocated to the CG (Figure 2).

### Subject characteristics and pulmonary function

Clinical data, persistent symptoms, comorbidities, medication use, and complete lung function are listed in Table 1. The mean age of the COVID-19 group was 40 years, 52% were men and had a body mass index higher than the CG (P<0.05). Fourteen patients required hospitalization in the ward with an average duration of six days. Fifteen patients stayed at home in self-isolation for an average of 10 days. In addition, 45% of survivors required low-flow oxygen supplementation during hospitalization. Regarding persistent symptoms, the main symptoms reported were fatigue, dyspnea, myalgia, hyposmia, dysgeusia, and headache. In this sense, patients who survived COVID-19 had more comorbidities (P<0.05), mainly hypertension, diabetes, and obesity. Additionally, it is important to note that in our sample 86% of COVID-19 survivors were sedentary compared to only 17% in the CG (P<0.05). We did not observe significant differences in the number of medications between the survivors and the CG.

**Table 1:** Clinical characteristics and pulmonary function of COVID-19 survivors and control group.

Variables	COVID-19 survivors (n=29)	Control group (n=18)	P
Age	40±11	38±13	0.62
Gender, n (%)			0.90
Female	14 (48)	9 (50)	
Male	15 (52)	9 (50)	
Weight (kg)	83±16	70±12*	0.007
Height (cm)	170±9	168±9	0.35
BMI (kg/m^2^)	28±4	25±3*	0.03
Duration of hospitalization (days)	6±3	11±1	1.12
Oxygen supplementation, n (%)	13 (45)	0 (0)	-
Oxygen supplementation (L)	3±2	0 (0)	-
Duration of self-isolation (days)	10±1	11±1	0.44
Smoking, n (%)	2 (7)	0 (0)	0.25
Ex-smoker, n (%)	4 (14)	0 (0)	0.09
Race, n (%)			0.27
White	22 (76)	16 (89)	
Brown	7 (24)	2 (11)	
Persistent symptoms, n (%)			
Fatigue	17 (59)	0 (0)*	<0.001
Dyspnea	7 (24)	0 (0)*	0.02
Cough	5 (17)	0 (0)	0.06
Dizziness	3 (10)	0 (0)	0.15
Memory loss	2 (7)	0 (0)	0.25
Myalgia	8 (28)	0 (0)*	0.01
Hyposmia	7 (24)	0 (0)*	0.02
Dysgeusia	8 (28)	0 (0)*	0.01
Headache	7 (24)	0 (0)*	0.02
Sleepiness	2 (7)	0 (0)	0.25
Weight loss	2 (7)	0 (0)	0.25
Comorbidity, n (%)			
Asthma	4 (14)	0 (0)	0.09
Depression	3 (10)	0 (0)	0.15
Hypertension	7 (24)	0 (0)*	0.02
Dyslipidemia	3 (10)	0 (0)	0.15
Diabetes	5 (17)	0 (0)*	0.06
Obesity	9 (31)	0 (0)*	0.009
Thyroid disease	1 (3)	0 (0)	0.42
Sedentary lifestyle	25 (86)	3 (17)*	<0.001
Medications, n (%)			
ACE inhibitors	2 (7)	0 (0)	0.25
Antidepressant	3 (10)	0 (0)	0.15
Anticoagulant therapy	2 (7)	0 (0)	0.25
Anti-hypertensive	4 (14)	0 (0)	0.09
Beta-blockers	1 (3)	0 (0)	0.42
Anti-hyperglycemic	5 (17)	0 (0)	0.06
Diuretics	5 (17)	0 (0)	0.06
Statins	3 (10)	0 (0)	0.15
Pulmonary function			
TV	4.05±1.1	4.20±1.0	0.63
FEV_1_ (L/s)	3.31±0.90	3.38±0.75	0.75
FEV_1_ (%)	96±15	98±11	0.64
FVC (L/s)	4.03±1.1	4.15±1.1	0.71
FVC (%)	98±15	105±11	0.13
FEV_1_/FVC (L/s)	0.81±0.06	0.80±0.04	0.72
TLC (L/s)	5.46±1.15	6.02±1.35	0.14
TLC (%)	91±20	102±17*	0.04
FRC (L/s)	3.22±1	3.60±1.4	0.27
FRC (%)	104±36	120±40	0.16
IC (L/s)	2.29±0.64	2.46±0.68	0.39
IC (%)	80±23	91±18	0.12
DLCO (mL)	21.51±5.95	26.78±7.39*	0.01
DLCO (%)	72±13	92±14*	<0.001
KCO (mL)	4.38±0.95	5.02±1*	0.03
KCO (%)	93±15	99±20	0.23
MIP (cmH_2_O)	74±30	88±39	0.18
MIP (% _pred_)	91±36	103±28	0.23
MEP (cmH_2_O)	94±31	123±50*	0.01
MEP (% _pred_)	90±27	123±50	0.07

Data are reported as means±SD or n (%). *P ≤0.05, unpaired Student's *t*-test was used for continuous variables and chi-squared test for categorical variables. BMI: body mass index; ACE: angiotensin enzyme inhibitor; TV: tidal volume; FEV_1_: forced expiratory volume in 1 s; FVC, forced vital capacity, TLC: total lung capacity; FRC: functional residual capacity; IC: inspiratory capacity; DLCO: carbon monoxide diffusing capacity; KCO: carbon monoxide transfer coefficient; MIP: maximum inspiratory pressure, MEP: maximum expiratory pressure.

In the assessment of pulmonary function, we did not find residual restrictive or obstructive changes and patients surviving COVID-19 had lower TLC (%) compared to the control group (P<0.05). However, the carbon monoxide diffusing capacity and carbon monoxide transfer coefficient were worse in the COVID-19 group compared to the CG (P<0.05). Regarding respiratory muscle strength, we observed a significant reduction in MEP (cmH_2_O) in the COVID-19 survivors' group (P<0.05), but we considered this of little clinical relevance (%) (P=0.07) (Table 1).

In CPET, COVID-19 survivors had lower values of load, *V̇*O_2_, *V̇*CO_2_, and peak RER compared with controls (P<0.05) (Table 2). Regarding ventilatory variables, we found that these patients had lower *V̇*_E_, BF, *P*_ET_CO_2_, Δ*P*_ET_CO_2_, and at peak exercise, an increase in *V̇*_E_/*V̇*CO_2_ slope was found compared to the CG (P<0.05). Although SpO_2_ was significant between the groups, it was not clinically relevant (both >95%). Additionally, the survivors group showed a worse chronotropic and CP response at the peak of the test and systemic blood pressure in the recovery period compared to the CG (P<0.05). It is noteworthy that factors such as peak leg effort score, SBP peak, and HR_max_ at peak exercise were the main criteria for test termination (Table 2).

**Table 2:** Between-group comparison of responses at peak and recovery from cardiopulmonary exercise testing.

Variables	COVID-19 survivors (n=29)	Control group (n=18)	P
Peak			
Work rate (W)	121±39	168±68*	0.005
Metabolic responses			
*V̇*O_2_ (mL/min)	1508±418	1979±781*	0.01
*V̇*O_2_ (mL·kg^−1^·min^−1^)	17.20±5	27.08±7*	<0.001
*V̇*O_2 pred_ (%)	61±13	86±19*	<0.001
*V̇*O_2_/WR (mL·min^−1^·W^−1^)	12.72±2.2	12±1.4	0.20
*V̇*CO_2_ (mL/min)	1581±495	2223±826*	0.002
RER _peak_	1.06±0.11	1.14±0.04*	0.01
Ventilatory responses			
*V̇*_E_ (L/min)	51±16	71±19*	0.001
BF (breaths/min)	33±7	40±10*	0.01
*V̇*_E_/*V̇*CO_2_ slope	35±9	28±2*	0.007
*P*_ET_CO_2 peak_ (mmHg)	32.87±4.1	36.59±3.4*	0.003
Δ *P*_ET_CO_2_ (mmHg)	1.96±2.8	3.97±1.6*	0.009
OUES	2.64±1.1	3.07±1.4	0.26
VP (mmHg)	6.31±2.15	7.00±1.12	0.22
SpO_2peak_ (%)	95±2	96±1*	0.04
Cardiovascular responses			
HR _peak_ (bpm)	145±24	164±17*	0.006
Δ HR _rest_ (bpm)	20±14	24±8	0.30
SBP _peak_ (mmHg)	205±24	199±22	0.41
SBP _rest_ (mmHg)	151±20	135±12*	0.004
DBP _peak_ (mmHg)	94±11	93±9	0.65
DBP _rest_ (mmHg)	86±8	80±3*	0.01
PD _peak_ (mmHg/bpm)	29698±514	32780±441*	0.04
Peak O_2_ pulse (mL/bpm)	10±3	12±4	0.22
CP (mmHg·mL^−1^·kg^−1^·min^−1^)	3130±101	4046±189*	0.03
Perception of symptoms			
Peak dyspnea score (0-10)	5±2	5±2	0.85
Peak leg effort score (0-10)	6±3	7±1*	0.05
Test interruption criteria			
Peak leg effort score	13 (45)	0 (0)*	0.001
Peak dyspnea score	4 (14)	0 (0)	0.09
Ventilation reserve	4 (14)	4 (22)	0.45
Heart rate reserve	5 (17)	3 (17)	0.95
Time	5 (17)	0 (0)	0.06
RER _peak_	13 (45)	15 (83)*	0.009
HR _máx_	3 (10)	0 (0)	0.15
SBP	6 (21)	0 (0)*	0.03
ECG	0 (0)	0 (0)	-

Data are reported as means±SD. *P ≤0.05, unpaired Student's *t*-test was used for continuous variables and chi-squared test for categorical variables. W: watts; *V̇*O_2_: oxygen uptake; *V̇*CO_2_: carbon dioxide output; RER: respiratory exchange ratio; *V̇*_E_: ventilation; BF: breathing frequency; *V̇*_E_/*V̇*CO_2_ slope: linear relation between minute ventilation and carbon dioxide output; *P*_ET_CO_2 peak_: end-tidal pressure for carbon dioxide; OUES: linear relation between oxygen uptake and minute ventilation; PV: ventilatory power; SpO_2_: peripheral saturation of oxygen; HR: heart rate; SBP: systolic blood pressure; DBP: diastolic blood pressure; DP: double product; CP: circulatory power; ECG: electrocardiogram.

**Figure 3:**
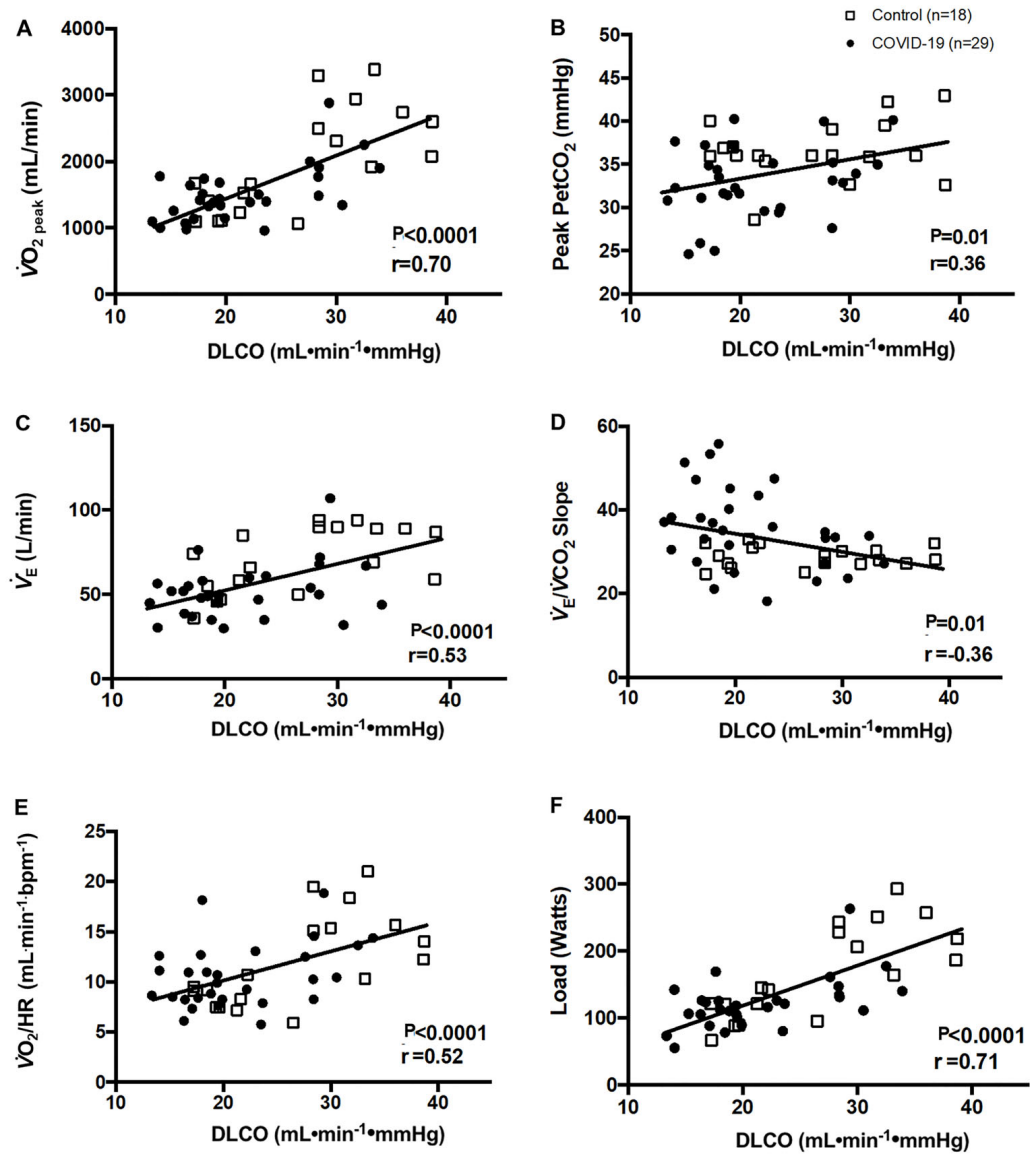
DLCO response profiles and their relationship with selected variables at cardiopulmonary exercise testing peak. Pearson's correlation analysis was performed to investigate correlations between variables. DLCO: carbon monoxide diffusing capacity; *V̇*O_2_: oxygen uptake; *P*_ET_CO_2 peak_: end-tidal pressure for carbon dioxide; *V̇*_E_: ventilation; *V̇*_E_/*V̇*CO_2_ slope: linear relation between minute ventilation and carbon dioxide output; *V̇*O_2_/HR: peak O_2_ pulse.

We also observed that reductions in *V̇*O_2_, *P*_ET_CO_2 peak_, *V̇*_E_, *V̇*_E_/*V̇*CO_2_ slope, peak O_2_ pulse, and load values at the peak of the test regardless of disease severity were all related to lower DLCO values (r=0.70 strong, r=0.36 moderate, r=0.53 moderate, r=0.36 moderate, r=0.52 moderate, and r=0.71 strong magnitude of the correlations, P<0.05, respectively) (Figure 3), demonstrating that the worse the DLCO, the lower the *V̇*O_2_, *P*_ET_CO_2 peak_, *V̇*_E_, workload, and *V̇*O_2_/load ratio changes and lower ventilatory efficiency, as represented by the *V̇*_E_/VCO_2_ slope in patients with COVID-19 of mild to moderate severity.

In addition, we observed that DLCO (mL·min^−1^·mmHg^−1^) and Δ*P*_ET_CO_2_ (mmHg) accounted for 37% of the variability in the peak *V̇*O_2_ (mL·kg^−1^·min^−1^) response in patients surviving COVID-19 (Table 3).

**Table 3:** Linear regression analysis to predict *V̇*O_2_ (mL·kg^−1^·min^−1^) of DLCO (mL) and Δ*P*_ET_CO_2_ (mmHg) in COVID-19 survivors.

Variables	β coefficient	Error	P value
DLCO (mL)	0.539	0.148	0.002
Δ*P*_ET_CO_2_ (mmHg)	-0.499	0.313	0.003

Adjusted R^2^=0.374; F=9.377 (P=0.001). *V̇*O_2:_ oxygen uptake_;_ DLCO: carbon monoxide diffusing capacity; Δ*P*_ET_CO_2:_ delta end-tidal pressure for carbon dioxide.

## Discussion

This is the first study to assess lung function and the response to CPET in less severe COVID-19 survivors compared with healthy controls. The main findings of this study were: 1) patients who survived COVID-19 had higher BMI, more comorbidities, were under more medications, and had more persistent symptoms after one month of infection; 2) the group of survivors had worse lung function compared to the CG, evidencing the significant involvement of DLCO; 3) all survivors of COVID-19 had reduced exercise capacity, achieved lower levels of exercise load and interrupted the exercise earlier, had higher *V̇*_E_/*V̇*CO_2_ and slopes, lower *V̇*_E_, CP, and SpO_2_ at peak test. In addition, we observed a depressed chronotropic response and blood pressure on CPET recovery; and 4) relationships between DLCO and low peak *V̇*O_2_, *P*_ET_CO_2 peak_, peak O_2_ pulse, *V̇*_E_, and load values, and a higher *V̇*_E_, *V̇*_E_/*V̇*CO_2_ slope, in mild-to-moderate severity COVID-19 survivors.

### Subject characteristics and pulmonary function

The post-viral infection syndrome, marked by the presence of persistent symptoms and comorbidities, was present in other coronavirus epidemics, such as SARS and MERS (30). In our study, post-COVID-19 manifestations were recorded in about 90% of recovered individuals, with a wide range of symptoms and conditions ranging from headache and myalgia to conditions such as depression; these manifestations persisted in all patients for at least 30 days since infection. The associations between the severity of COVID-19 were also observed in hypertension, cardiovascular disease, and diseases of the respiratory system (9).

Overall, critically ill patients were older and had a higher number of comorbidities and longer hospital stays (31). However, we found that younger patients (under 40 years) and survivors of less severe cases after one month of infection had a significant burden of comorbidities. In this sense, 89% of the adult survivors of COVID-19 evaluated were sedentary. Raman et al. (4) and Bellan et al. (6) highlight that emotional factors such as anxiety and depression may be related to physical inactivity after hospitalization for COVID-19. In addition, the decrease in physical activity levels induced by confinement and the increase in sedentary behavior can lead to a rapid deterioration of health (32). Even short-term inactivity (1-4 weeks) has been linked to detrimental effects on cardiovascular function and structure and increased cardiovascular risk factors (32). If the prevalence of chronic conditions caused by inactive lifestyles were lower, the impacts in the post-COVID-19 period could be minimized (5).

It is noteworthy that in our study none of the hospitalized patients had signs of severity during hospitalization (Table 4). Another important point is the length of stay. Previous studies (1,9) reported prolonged periods of bed rest, in addition to a greater mix of different severities of COVID-19 and the absence of a CG, which makes interpretation of the true impact of COVID-19 after the infection phase difficult. To eliminate confounding factors, we only included mild-to-moderate cases that remained hospitalized or quarantined on average for the same period (Table 1). In addition, all hospitalized patients received respiratory and physical therapy during their hospital stay.

**Table 4:** Blood gas analysis and blood count in patients with COVID-19 on hospital admission.

Variables	COVID-19 survivors (n=14)
Arterial blood gas analysis	
pH	7.45±0.03
PaO_2_ (mmHg)	79±13
PaCO_2_ (mmHg)	37±4
HCO_3_ (mmHg)	25±3
Lactate (mmol/L)	0.9±0.8
SpO_2_	92±2
Blood count	
Hemoglobin (g/dL)	14±1.3
Leukocytes (N/mm^3^)	7810±2654
Platelets (µL)	236.000±77113
Lymphocytes (N/mm^3^)	4825±817
Neutrophils (N/mm^3^)	5306±2197
Eosinophil (µL)	100±118
C-reactive protein (mg/L)	4.13±6.3
Troponin-I (ng/L)	0.014±0.53
Total CK (U/L)	44±23
Creatinine (mg/dL)	0.85±0.24
D-dimer (mcg/mL)	0.76±0.44

Data are reported as means±SD. SpO_2_: peripheral saturation of oxygen; CK: creatine kinase; PaO_2_: oxygen blood pressure; PaCO_2_: carbon dioxide arterial pressure; HCO_3_: base excess.

We did not find significant reductions in VT, FEV_1_, and FVC in COVID-19 survivors after one month of hospitalization compared to the control group. Recently, Mo et al. (33) evaluated 110 patients who recovered from severe COVID-19, and a pulmonary function test was performed on the day before or on the day of hospital discharge. This group reported changes in FEV_1_ in 13% of cases, FVC in 10%, and FEV_1_/FVC in 4.5%. In addition, the authors point out that the percentage value of TLC was 79% of the predicted value for severe cases of pneumonia caused by COVID-19. In the results of the present study, involvement of TLC was greater in survivors (TCL 91% of predicted) compared to the CG (TLC 102% of predicted). According to Raman et al. (4), 13% of critically ill patients exhibited FVC abnormalities within 2-3 months after hospital admission. It is noteworthy that published studies lack sample homogeneity, with important differences in age, COVID-19 severity, and absence of a control group for comparisons. In an unprecedented way, we found that milder manifestations of SARS-COV-2 infection did not cause chronic obstructive and/or restrictive changes in our patients after one month.

DLCO deficiency is an early abnormality in patients who survived COVID-19. In our study, DLCO abnormalities occurred in most surviving patients, the data indicated impaired intra-alveolar diffusion pathways. Meo et al. (34) reported that SARS and COVID-19 had similar biological and clinical characteristics. Previous studies of SARS survivors have shown that impaired DLCO was the most common abnormality in the post-infection period, ranging from 15 to 43% of patients ([Bibr B34] [Bibr B35] 3436). Our results were consistent with these findings. We observed changes in DLCO in 65% of patients, which reached an average of 72% of the predicted values for DLCO. Autopsy in patients who died of COVID-19 showed different degrees of destruction of the alveolar structure and interstitial pulmonary fibrosis (37). Huang et al. (7) also reported that more than 50% of patients had DLCO of less than 80% of the expected 30 days after hospital discharge, and 30% of patients had severe or critical disease.

Bellan et al. (6) reported that of the 219 patients evaluated, 51% had values lower than 80% of the predicted value and 15% of the volunteers had values lower than 60% of the predicted DLCO four months after hospital discharge. The authors emphasize that female gender, a history of comorbidities, and oxygen supplementation in the acute phase were associated with a reduction in DLCO (6,7). In this sense, we observed that 45% of our patients required oxygen supplementation durian hospital stay, in addition to having a greater number of associated comorbidities. On the other hand, according to the authors, chronic obstructive pulmonary disease and ICU admission were associated with losses in lung volumes and capacities (6). However, although we have not evaluated critical cases, our results contribute to the understanding of a patient population that has been poorly studied and that presents significant alterations in the capacity of carbon monoxide diffusion. However, a study by Zhao et al. (38) reported that only 9 of 55 patients (16%) had a DLCO of 80% of the predicted 3 months after hospital discharge. We sought to observe this behavior in a younger population with mild-to-moderate manifestations of COVID-19 after a short period since diagnosis compared to the control group; a methodological profile of the study needs further investigation.

We observed a reduction in expiratory muscle strength in the group of survivors of COVID-19. This finding suggests muscle wasting secondary to a catabolic state induced by manifestations of viral infection and potentially aberrant systemic inflammation (39). However, we emphasize that none of our patients suffered from prolonged immobility and severe or critical manifestations during the infection, which could justify the maintenance of normal values in mean MEP in COVID-19 survivors.

### Cardiopulmonary exercise testing

According to Ong et al. (40), 41% of SARS survivors had limitations in exercise tolerance months after infection. In our study, we observed a lower value of peak *V̇*O_2_ (mL·kg^−1^·min^−1^) and *V̇*O_2pred_ (%) in all COVID-19 survivors. Our results have characteristics similar to the results of Raman et al. (4), who evaluated moderate and severe patients, and Rinaldo et al. (15) in a cross-sectional study of 18 subjects with mild-to-moderate disease out of a total of 78 patients: both showed a significant decrease in aerobic capacity. However, both studies (4,15) included patients of advanced age and different severities with a prolonged hospital stay. We found that COVID-19 survivors showed a significant reduction in aerobic capacity, despite not suffering from immobility during infection and having a lower mean age. In addition, the authors emphasize that the degree of systemic inflammation could limit exercise capacity (4). In the present study, the patients did not show aberrant increases in inflammatory markers during COVID-19 despite a poorer performance on CPET after infection (Table 4).

In the present study, the *V̇*_E_/*V̇*CO_2_ slope, a measure of ventilatory efficiency, was worse in COVID-19 survivors. In addition, peak SpO_2_ and Δ*P*_ET_CO_2_ were lower in that group. Raman et al. (4) and Gao et al. (13) found an increase in the *V̇*_E_/*V̇*CO_2_ slope in older patients hospitalized for a longer time. The authors (4,13) highlight that the finding was probably due to peripheral factors from exposure to steroids and prolonged hospitalization. Differing from previous studies, we found an abnormal behavior in younger patients with lower severity of COVID-19 who remained hospitalized for a shorter time. Furthermore, the *P*_ET_CO_2_ values observed in our study were similar to that of older patients with a mean of 30 days of hospitalization (14). This indicates an important change in the corresponding function of ventilation and perfusion in the pulmonary system and cardiac function in adult survivors of both non-severe and severe COVID-19 (27).

We found that CP, a potent marker of systolic function, was significantly lower in COVID-19 survivors (25). In addition, these patients had a lower O_2_ pulse and depressed chronotropic response at the peak of the test, in addition to a worse systemic blood pressure response in CPET recovery. It is important to note that many patients discontinued CPET early due to lower limb fatigue and SBP values. These findings suggest that muscle loss, secondary to a mild catabolic state induced by the disease and a higher BMI, can lead to reduced exercise capacity, in addition to depressed chronotropic response in these survivors (1). Furthermore, we can speculate that such autonomic derangement may be associated with an abnormal distribution of cardiac output to exercising muscles, thus contributing to low peripheral oxygen extraction (14).

We found that reduced DLCO values were associated to worse *V̇*O_2_, peak *P*_ET_CO_2_, peak O_2_ pulse, load values and slope values *V̇*_E_, *V̇*_E_/*V̇*CO_2_ (P<0.05). The magnitude of the correlations was moderate to high (Figure 3). Other studies (4,13-15) reported reduced exercise capacity in moderate-to-severe COVID-19 survivors. Although they showed slight reductions in lung function, the authors emphasize that this condition could not explain the impairment of exercise tolerance. Furthermore, in the study by Raman et al. (4), serum markers of inflammation (r=0.32, P=0.02) and severity of illness were correlated with exercise tolerance.

Rinaldo et al. (15) did not find relationships to discriminate peak *V̇*O_2_ between survivors with and without preserved aerobic capacity. This profile of functional limitation after COVID-19 is similar to that found in SARS survivors (34). Additionally, Baratto et al. (14) states that low peak *V̇*O_2_ values were associated with CaO_2_ and hemoglobin. Thus, the literature indicates the magnitude of multisystem involvement and its repercussions on patients who survived COVID-19 in severe and critical cases. Given our findings, we emphasize that survivors of mild cases deserve attention, since these subjects had important limitations, despite showing mild COVID-19 signs and symptoms. In this sense, CPET can contribute to the identification of the main limiting factors during physical exertion and assist health professionals to develop effective rehabilitation strategies, with the objective of reversing cardiorespiratory and functional changes in COVID-19 survivors (13).

### Study limitations

Our study had limitations inherent to its cross-sectional nature, such as being carried out in a single city center. However, the procedures adopted for the treatment of COVID-19 and the rehabilitation protocol were similar between the institutions, where the study was carried out, providing reliability regarding the impact of in-hospital rehabilitation on the functional capacity of our patients. Secondly, our data cannot be extrapolated to the general population of patients affected by COVID-19, since a large portion of severe cases are individuals of advanced age, who are known to have more musculoskeletal dysfunction and more compromised immunity. In the present study, we excluded the elderly and, consequently, the most severe cases that culminate in longer hospital stays and significantly impact functional capacity. Our findings are restricted to the middle-aged and younger population, with mild to moderate cases of the disease and with a length of stay of less than 10 days, thus eliminating the bias of length of stay in the impact on functionality. We observed a high rate of comorbidities that could influence our results, mainly cardiovascular comorbidities on DLCO. However, we emphasize that this is a common feature among COVID-19 survivors regardless of disease severity. Finally, we did not perform a functional assessment at the time of hospital discharge, but all volunteers were monitored during hospitalization and contacted by telephone calls to monitor their health conditions on day seven and day fourteen after the illness.

This is the first study to comprehensively assess the short-term effects of mild COVID-19 in younger adult patients, finding depressed cardiopulmonary responses to maximal exercise and reduced DLCO. Our results highlight the need to develop a multidisciplinary approach for the clinical care of mild COVID-19 patients. One month after recovery from COVID-19 infection, we observed a high burden of persistent symptoms, changes in lung function, and low CRF, which bring important implications for individuals who must return to work after infection. However, further large-scale studies investigating the long-term effects of COVID-19 in young adults are needed to fully understand the burden of chronic disease among survivors of SARS-CoV-2 infections and the long-term implications on lung function and exercise tolerance. We continued to monitor these matters for a year seeking to understand the changes imposed by COVID-19.
